# Kynurenine Pathway as a New Target of Cognitive Impairment Induced by Lead Toxicity During the Lactation

**DOI:** 10.1038/s41598-020-60159-3

**Published:** 2020-02-21

**Authors:** Daniela Ramirez Ortega, Paulina Ovalle Rodríguez, Benjamín Pineda, Dinora F. González Esquivel, Lucio Antonio Ramos Chávez, Gustavo I. Vázquez Cervantes, Gabriel Roldán Roldán, Gonzalo Pérez de la Cruz, Araceli Díaz Ruiz, Marisela Méndez Armenta, Jaime Marcial Quino, Saul Gómez Manzo, Camilo Ríos, Verónica Pérez de la Cruz

**Affiliations:** 1 Laboratorio de Neurobioquímica y Conducta, Departamento de Neuroquímica, Instituto Nacional de Neurología y Neurocirugía Manuel Velasco Suárez, S.S.A., México D.F, 14269 México; 2 Laboratorio de Neuroinmunología, Instituto Nacional de Neurología y Neurocirugía Manuel Velasco Suárez, S.S.A., México D.F, 14269 México; 3 Laboratorio de Neurobiología de la Conducta, Departamento de Fisiología, Facultad de Medicina, Universidad Nacional Autónoma de México, Ciudad de México, 04510 México; 4 Facultad de Ciencias, Departamento de Matemáticas, UNAM. Circuito Exterior, CU, 04510 Ciudad de México, México; 5 Laboratorio de Neuropatología Experimental, Instituto Nacional de Neurología y Neurocirugía, Manuel Velasco Suárez, S.S.A., México D.F, 14269 México; 6 CONACYT- Instituto Nacional de Pediatría, Secretaría de Salud, Ciudad de México, 04530 México; 7 Laboratorio de Bioquímica Genética, Instituto Nacional de Pediatría, Secretaría de Salud, México City, 04530 México

**Keywords:** Neurochemistry, Environmental impact, Risk factors

## Abstract

The immature brain is especially vulnerable to lead (Pb^2+^) toxicity, which is considered an environmental neurotoxin. Pb^2+^ exposure during development compromises the cognitive and behavioral attributes which persist even later in adulthood, but the mechanisms involved in this effect are still unknown. On the other hand, the kynurenine pathway metabolites are modulators of different receptors and neurotransmitters related to cognition; specifically, high kynurenic acid levels has been involved with cognitive impairment, including deficits in spatial working memory and attention process. The aim of this study was to evaluate the relationship between the neurocognitive impairment induced by Pb^2+^ toxicity and the kynurenine pathway. The dams were divided in control group and Pb^2+^ group, which were given tap water or 500 ppm of lead acetate in drinking water ad libitum, respectively, from 0 to 23 postnatal day (PND). The poison was withdrawn, and tap water was given until 60 PND of the progeny. The locomotor activity in open field, redox environment, cellular function, kynurenic acid (KYNA) and 3-hydroxykynurenine (3-HK) levels as well as kynurenine aminotransferase (KAT) and kynurenine monooxygenase (KMO) activities were evaluated at both 23 and 60 PND. Additionally, learning and memory through buried food location test and expression of KAT and KMO, and cellular damage were evaluated at 60 PND. Pb^2+^ group showed redox environment alterations, cellular dysfunction and KYNA and 3-HK levels increased. No changes were observed in KAT activity. KMO activity increased at 23 PND and decreased at 60 PND. No changes in KAT and KMO expression in control and Pb^2+^ group were observed, however the number of positive cells expressing KMO and KAT increased in relation to control, which correlated with the loss of neuronal population. Cognitive impairment was observed in Pb^2+^ group which was correlated with KYNA levels. These results suggest that the increase in KYNA levels could be a mechanism by which Pb^2+^ induces cognitive impairment in adult mice, hence the modulation of kynurenine pathway represents a potential target to improve behavioural alterations produced by this environmental toxin.

## Introduction

Lead (Pb^2+^) is one of the main environmental neurotoxins that represents a health problem due to its multiple industrial, domestic, medical and technological applications^1^. Because its wide distribution, Pb^2+^ exposure can occur via eating, drinking or inhalation. This metal can cross placental and blood brain barrier (BBB) and also it is accumulated in several tissues, such as the lungs, liver, kidneys, spleen, brain and bones^2^. It has been reported that the central nervous system (CNS) is a target of lead exposure^3–5^. The developing brain is especially vulnerable to Pb^2+^ neurotoxicity since this heavy metal crosses through the placenta during pregnancy and also is able to pass into milk during the lactation period, reaching the developing brain impacting its functionality and morphology^6,7^. There are reports showing that even low doses of Pb^2+^ cause cognitive and motor functions impairment in humans and animal models^8^, mainly when the exposure occurs during brain development, where there are intense cellular proliferation, differentiation and synaptogenesis.

In this regard, it has been shown that Pb^2+^, at low micromolar concentrations, inhibits in an age-dependent manner the N-methyl-D-aspartate receptor (NMDAr) activation, being more harmful in the neonatal than in adult brain and producing long-term cognitive impairment^3,9,10^. During the early postnatal life, the NMDAr activation is essential for the development of neuronal networks; its inhibition in this period leads to severe neuromorphological and behavioral deficits^11–15^. Concerning to this, kynurenine pathway (KP) constitutes the main catabolic route of the essential amino acid tryptophan in mammals, which produces important metabolites with redox and neuroactive properties^16^. Specifically, kynurenic acid (KYNA) has been involved in brain development^17^ and cognitive process^18^ since it is the unique endogenous antagonist of NMDA receptor and a negative allosteric modulator of alpha7-nicotinic receptors. It has been suggested that the high KYNA levels found in fetal brain as well as its rapid decline during the postnatal period, are involved in NMDAr modulation, necessary for the normal brain development^19,20^. In this context, it has been shown that the fluctuation in brain KYNA levels can cause impairment in working memory, sensorimotor gating and in the attention process in adult rodents^21–23^.

Numerous publications have recently demonstrated that both Pb^2+^ exposure as well as KYNA levels fluctuation during the pre- and postnatal period, lead to cognitive impairment. However, there are no studies that have linked them, even knowing that there are several assumptions and converging findings shared: (1) kynurenine pathway is modulated by redox and inflammatory environment, which are altered by Pb^2+^ toxicity, (2) both Pb^2+^ and KYNA are able to inhibit NMDAr, (3) the developing brain is especially susceptible to lead toxicity as well as to KYNA levels fluctuations and (4) Pb^2+^ and high KYNA levels at early life induce behavioral alterations. Hence, to explore KP alterations as a new toxic mechanism by which Pb^2+^ induces cognitive impairment, represents a novel approach to modulate tryptophan catabolism as a therapeutic strategy. Therefore, in this work, we evaluated whether Pb^2+^ exposure, during the lactation period, induces changes in KP metabolites that correlate with long-term cognitive impairment. For this purpose, the dams received lead acetate in drinking water from 0 to 23 PND (lactation period). Immediately after weaning or one month after Pb^2+^ exposure, behavioral performance, KP metabolites and enzyme activity as well as morphological changes were analyzed. Our results support that KP metabolites, particularly the high levels of KYNA, are correlated with long-term cognitive impairment induced by Pb^2+^ during the lactation period.

## Results

### Brain Pb^2+^ levels in mice after 23 days of exposure

**Figure 1:**
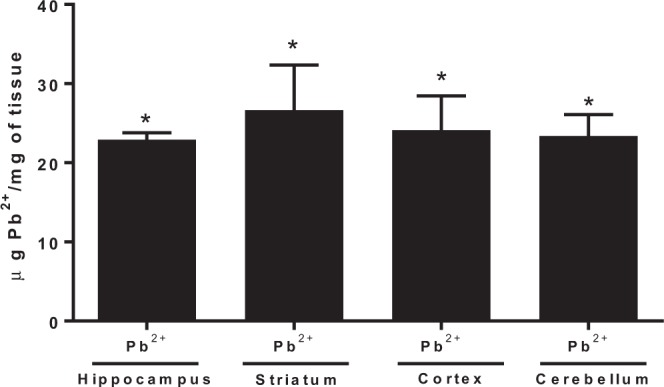
Lead concentration in different brain regions of mice at 23 PND, after Pb^2+^ exposure during lactation period. Determinations were made in hippocampus, striatum, cortex and cerebellum of mice. Data are mean ± S.E.M. T-test and Mann-Whitney test were used for comparisons, *P < 0.01 vs. each respective brain region control, n = 5 per group.

The concentration of Pb^2+^ (500 ppm) used in this study had no observable effects on mortality of the litters and had no observable adverse physical effects. Figure 1 shows the reached levels of Pb^2+^ in each brain region evaluated after 23 days of treatment (lactation period). The levels of Pb^2+^ found in hippocampus, striatum, cortex and cerebellum were 23 ± 0.8, 26.7 ± 5.6, 24.1 ± 4.4, 23.4 ± 2.8 µg Pb^2+^/mg of tissue, respectively in mice treated with Pb^2+^during lactation period. No detectable levels were found in control group.

### Locomotor activity alterations induced by lead exposure

**Figure 2:**
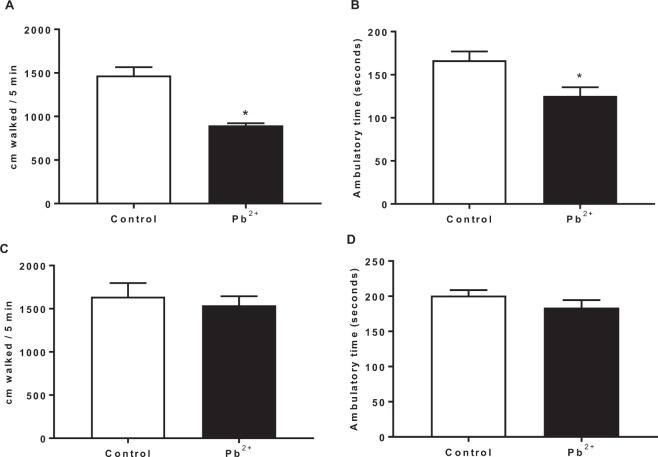
Locomotor activity of mice at 23 PND (**A,B**) and at 60 PND (**C,D**) in open field after Pb^2+^ exposure in drinking water during lactation period. Motor activity was assessed as the total distance walked (**A**,**C**) and the ambulatory time (**B**,**D**). Data are mean ± S.E.M. *P < 0.01 vs. control, T-test and Mann-Whitney test. N = 10–15 per group and per age.

First, we examined the immediate effect of lead treatment during lactation period (23 PND) on locomotor activity of mice (Fig. 2). We found that the total distance traveled (Fig. 2A) as well as the ambulatory time (Fig. 2B) significantly decreased in the mice of Pb^2+^ group compared to control group. However, when long-term effect (60 PND) was determined, no changes were found between control group and Pb^2+^ group (Fig. 2C,D).

### Lead exposure during the lactation period induce memory impairment

**Figure 3:**
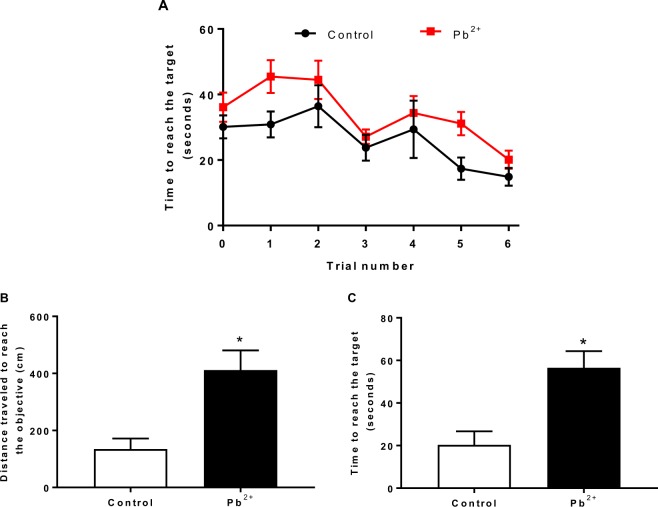
Long-term memory evaluation after Pb^2+^ exposure during lactation period. Memory impairment in adult mice was evaluated through the distance traveled (**B**) and the time to reach target (**C**) 24 h after acquisition phase (**A**). Data are mean ± S.E.M. *P < 0.01 vs. control, T-test and Mann-Whitney test. N = 9–15 per group.

Learning and memory of mice (60 PND) treated with Pb^2+^ during the lactation period were examined through the buried food location test (Fig. 3). Training phase is shown in panel A. Pb^2+−^ group mice showed the same learning pattern that control group, in which they learned to reach the target. These data suggest that Pb^2+^ did not affect the acquisition phase. However, when long-term memory was evaluated 24 h after acquisition, both, the distance travelled as well as the time to reach the target were higher in Pb^2+^ group than in control group, suggesting memory consolidation impairment in the Pb^2+−^ group.

### Redox environment alterations and cellular dysfunction induced by Pb^2+^ treatment

Reactive oxygen species (ROS), lipid peroxidation and GSH levels were evaluated in Pb^2+−^ treated mice at 23 and 60 PND (Table 1). As expected, ROS levels and lipid peroxidation were higher (50–70% and 40–110%, respectively) in Pb^2+^ group than in controls, in all brain regions determined at 23 PND. However, the high levels of ROS were not observed at 60 PND in the group treated with Pb^2+^. Lipid peroxidation was higher in hippocampus and cerebellum (~75% and 120%, respectively) of mice treated with Pb^2+^ during lactation period compared with control group. The GSH levels decreased around 20% in cerebellum of mice under treatment with Pb^2+^ compared to control at both times evaluated. Cellular function determined by MTT reduction, decreased around 40–50% in all brain regions of mice treated with Pb^2+^ compared to controls at both times evaluated.

**Table 1:** Effects of Pb^2+^ exposure on brain redox environment.

	23 PND							
	Hippocampus		Striatum		Cortex		Cerebellum	
	C	Pb^2+^	C	Pb^2+^	C	Pb^2+^	C	Pb^2+^
ROS (mg of DCF/mg of protein)	0.33 + 0.03	0.57 + 0.05*	0.85 + 0.08	1.46 + 0.08*	1.30 + 0.09	1.52 + 0.03*	0.79 + 0.06	1.20 + 0.07*
Lipid peroxidation (µmoles of MDA/mg of protein)	0.037 + 0.001	0.077 + 0.008*	0.042 + 0.004	0.069 + 0.001*	0.026 + 0.002	0.036 + 0.001*	0.021 + 0.001	0.046 + 0.004*
MTT (% vs. Control)	100 + 5.1	65.77 + 4.77*	100 + 4.2	68.75 + 4.75*	100 + 5.0	48.05 + 5.55*	100 + 8.4	60.11 + 5.61*
GSH (nmol/g of tissue)	2689.72 + 84.72	2630.15 + 89.85	2485.13 + 184.87	2531.12 + 278.88	2541.32 + 51.32	2603.96 + 88.96	2742.99 + 52.99	2548.51 + 48.51*
	60 PND							
ROS (mg of DCF/mg of protein)	0.86 + 0.084	0.93 + 0.068	1.22 + 0.203	1.69 + 0.31	0.35 + 0.017	0.27 + 0.051	0.21 + 0.011	0.19 + 0.021
Lipid peroxidation (µmoles of MDA/mg of protein)	0.42 + 0.057	0.737 + 0.143*	0.464 + 0.071	0.7 + 0.1	0.26 + 0.028	0.25 + 0.055	0.110 + 0.01	0.25 + 0.07*
MTT (% vs. Control)	100 + 4.2	50.76 + 5.74*	100 + 16	46.54 + 3.46*	100 + 4.1	53.38 + 3.62*	100 + 7.2	75.76 + 5.24*
GSH (nmol/g of tissue)	2583.66 + 111.34	2573.11 + 131.89	2146.92 + 233.08	2237.48 + 137.52	2323.63 + 66.37	2246.58 + 153.42	2668.05 + 91.95	2256.63 + 83.37*

Data were obtained in different cerebral regions: hippocampus, striatum, cortex and cerebellum at 23 and 60 PND. Data are mean ± S.E.M. T-test and Mann-Whitney test were used for comparisons, *P < 0.01 vs. each respective brain region control, n = 5–8 per group.

### Kynurenine Pathway alterations by Pb^2+^ exposure during lactation period of mice

**Figure 4:**
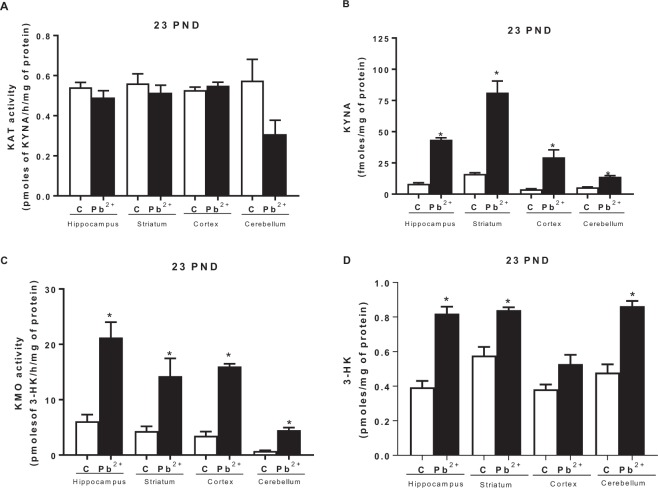
Kynurenine pathway alterations induced by Pb^2+^. (**A**) KAT II activity, (**B**) KYNA levels, (**C**) KMO activity and (**D**) 3-HK levels were evaluated in hippocampus, striatum, cortex and cerebellum of mice at 23 PND, after Pb^2+^ exposure during lactation period. Data are mean ± S.E.M. *P < 0.01 vs. each respective brain region control, T-test and Mann-Whitney test. N = 9 per group.

To evaluate the impact of Pb^2+^ exposition on KP during lactation period, we analyzed both arms of the pathway: by one side, kynurenine aminotransferase (KAT II) activity as well KYNA levels, and on the other side, kynurenine monooxygenase (KMO) activity as well 3-HK levels, immediately after removing drinking water with Pb^2+^ (23 PND) and also at 60 PND, to examine long-term effects (Fig. 4). The KAT II activity (the main enzyme to produce KYNA) did not show changes in Pb^2+^ group compared to control group (Fig. 4A). However, KYNA levels showed an increase in each cerebral region evaluated (Fig. 4B, 528 ± 18, 500 ± 58, 752 ± 150 and 255 ± 17% for hippocampus, striatum, cortex and cerebellum, respectively) compared with its respective brain region control (p < 0.001). KMO activity and 3-hydroxykynurenine levels at 23 PND showed a significant increase (p < 0.05) in all brain regions analyzed (Fig. 4C,D, respectively).

**Figure 5:**
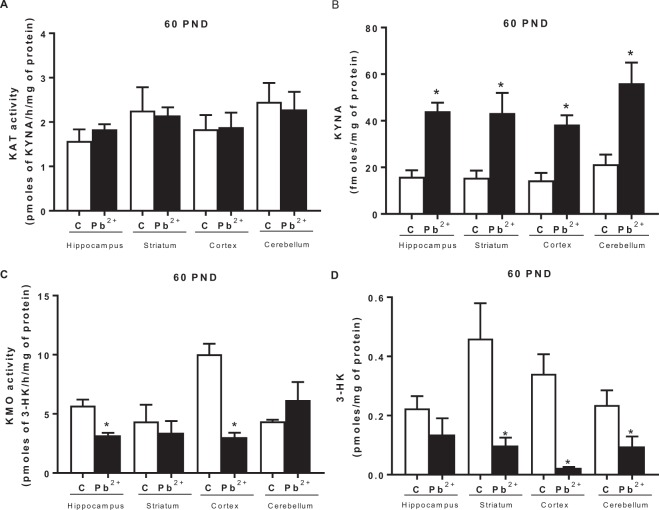
Long-term alterations on kynurenine pathway generated by Pb^2+^ treatment during lactation period of mice. (**A**) KAT II activity, (**B**) KYNA levels, (**C**) KMO activity and (**D**) 3-HK levels were evaluated in hippocampus, striatum, cortex and cerebellum of mice at 60 PND. Data are mean ± S.E.M. *P < 0.01 vs. each respective brain region control, T-test and Mann-Whitney test. N = 8 per group.

**Figure 6:**
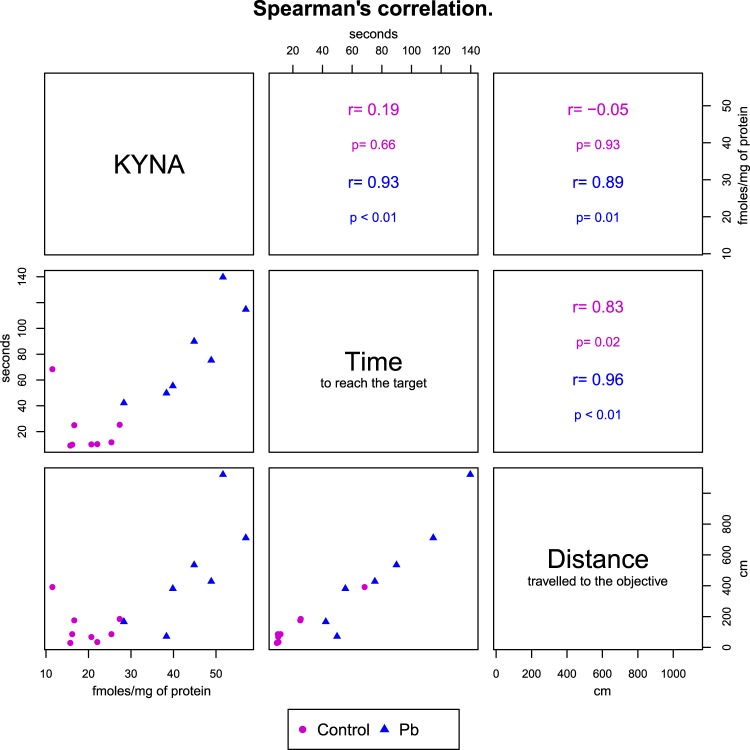
The lower triangular matrix contains the scatterplot for each pair of variables for both groups (control and Pb^2+^). The upper triangular matrix contains the Spearman’s rank correlation coefficient (r) and its associated p-value (p) calculated for each group.

When long-term effects were evaluated, we found no changes in KAT II activity (Fig. 5A); however, KYNA levels were still high (around double compared to control) in all the cerebral regions evaluated (p < 0.01; Fig. 5B). KMO activity decreased significantly in hippocampus and cortex (Fig. 5C), and 3-HK levels decreased significantly in striatum, cortex and cerebellum (Fig. 5D) of mice treated with Pb^2+^ during lactation period compared to control group (p < 0.01). Additionally, we found that KYNA levels determined at 60 PND, correlated with the cognitive impairment assessed by total distance traveled to reach the objective and time to reach the target (Fig. 6), suggesting that KYNA is related with the cognitive impairment induced by Pb^2+^ treatment during lactation period. No correlation was found between 3-HK levels and cognitive impartment parameters.

### Lead treatment decreases the number of neurons and changes the cellular proportion of KMO**+** and KAT II**+** brain cells

**Figure 7:**
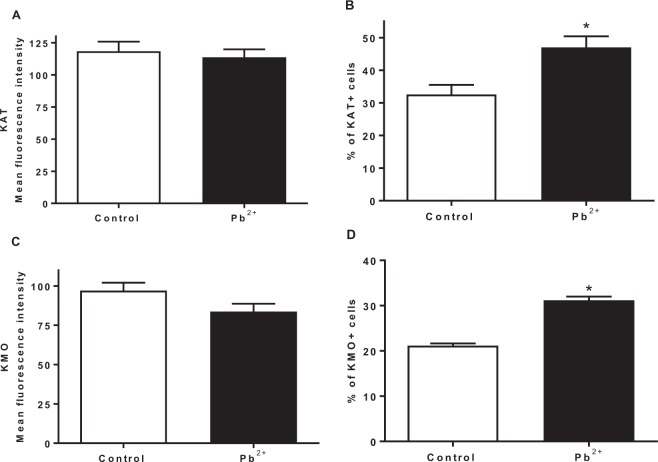
Effect of Pb^2+^ treatment during lactation on the proportion of KAT II + and KMO + brain cells. Expression of KAT II (**A**) and KMO (**C**) was determined in the brain of mice at 60 PND. Number of positive cells to KATII (**B**) and KMO (**D**) were evaluated in the same samples. Data are mean ± S.E.M. *P < 0.05 vs. each respective brain region control, T-test and Mann-Whitney test.

**Figure 8:**
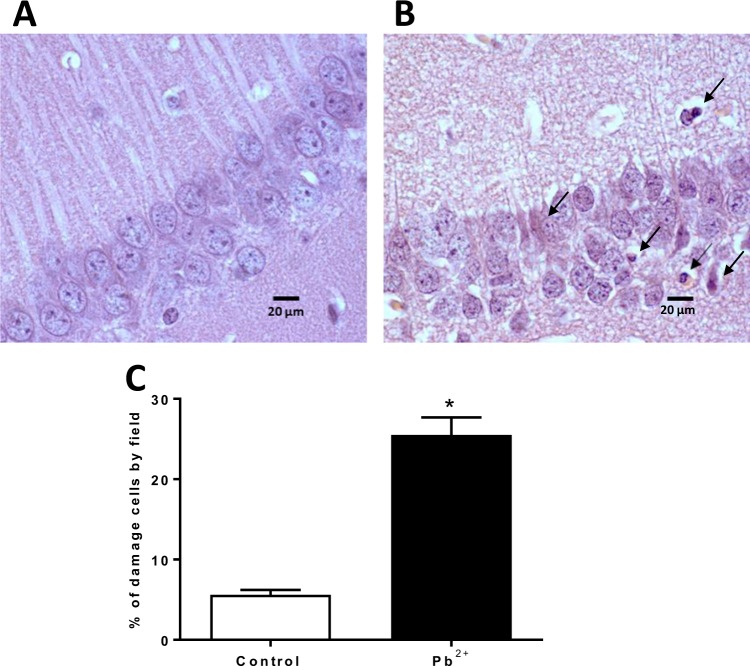
Representative photomicrographs of hematoxylin-eosin-stained hippocampus sections (100x) of control and Pb^2+^ groups. (**A**) Control group, normal cells exhibited round-shaped and pale-stained nuclei. (**B**) Pb^2+^ group, dead cells exhibited a shrunken cytoplasm with pyknotic nuclei (black arrows). (**C**) Percentage of cellular damage in the hippocampal region of mice exposed to Pb^2+^. Values are expressed as the percentage of damaged cells counted in ten fields per area, in three slides per mouse. *P < 0.001 vs. control, T-test and Mann-Whitney test.

After evaluating the KP metabolites and observing that KYNA levels were higher in Pb^2+^ group but not KAT II activity, we decided to evaluate the expression of KAT II in the positive brain cells, both, in control group as Pb^2+^ group at 60 PND. Figure 7A,C show the expression of KAT II and KMO in full brain, respectively; no changes in the mean fluorescence intensity were found. However, the number of positive cells from both KAT II and KMO increased significantly, indicating that the cellular proportion that express these enzymes changed (Fig. 7B,D, respectively) with Pb^2+^. This would mean that Pb^2+^ treatment during the lactation period induces neuronal cell death and in consequence the proportion of brain cells changes. Figure 8A,B show the representative histological micrographs of the hematoxylin-eosin stained hippocampus of control and Pb^2+^ groups (60 PND). The majority of neurons in the hippocampal CA1 subfield of mice in the Pb^2+^ group appeared shrunken with eosinophilic cytoplasm, pyknotic nuclei and slight interstitial edema; while in control group there was no evidence of neuronal damage, cells exhibited round-shaped, with a lack of eosinophilic cytoplasm or evident pyknotic nuclei. The number of surviving neurons in Pb^2+^ group was significantly lower than in the control group (P < 0.01; Fig. 8C).

## Discussion

In the present work, we studied the effect of Pb^2+^ exposition in mice, during lactation period, in KP and its relationship with long-term cognitive impairment induced in this toxic model. Lead is a heavy metal considered one of the most widespread and harmful environmental toxin^24^. Many mechanisms have been involved in Pb^2+^ toxicity and some of them converge as oxidative stress, inflammation, energetic alterations, calcium homeostasis perturbations, among others^25–28^. These factors can influence the tryptophan catabolism trough KP, which produces metabolites with neuroactive and redox properties that affect brain function^29–31^. Alterations in KP during brain development lead to cognitive impairment in adulthood^32^. The KP relationship with Pb^2+^ toxicity and with the cognitive impairment induced by this metal, could begin to be recognized by the fact that Pb^2+^ generates an oxidant environment that can cause fluctuations in the KP metabolites having repercussion in the cognition^33^. However, this is the first study relating increased KYNA levels and Pb^2+^ exposure.

In this study, we used a model of Pb^2+^ exposure in mice, during lactation period, knowing that the brain in prenatal and early postnatal periods undergoes rapid growth and is very sensitive to environmental pollutants, including Pb^2+^, as well as taking in consideration that KP plays an important role in brain development^19,34^. First, Pb^2+^ concentration was determined in different brain regions founding that it was deposited in CNS and as was expected, the redox environment and cellular function were also affected after Pb^2+^ exposure during lactation of mice at 23 PND. These results indicated that the oxidant cellular environment may induce tryptophan catabolism, mainly through KP. According to this, high levels of KYNA and 3-HK, as well as increased KMO activity were found in the brain of Pb^2+^ group at 23 PND. However, no changes were found when the KAT II activity -the main enzyme responsible of KYNA production- was evaluated in these mice, indicating that the high levels of KYNA were produced by no canonical routes (^30,35^. In this context, new pathways of KYNA production have been described, one of them involves the interaction of reactive oxygen species with L-kynurenine (the precursor of KYNA and 3-HK) or tryptophan^30,35,36^. According to this background, the high brain levels of ROS that were found, under our experimental conditions, can contribute directly to KYNA production but also can promote the augment in the KP, since indoleamine-2, 3-dioxygenase (IDO, enzyme responsible of the first step of KP, via tryptophan cleavage) and KMO are modulated by superoxide anion.

Considering these premises and findings, which indicate that both, Pb^2+^ and KP fluctuations, during early postnatal life, impact on cognition in the adulthood, the next step was to evaluate if fluctuations in KP metabolites levels induced by Pb^2+^ toxicity during lactation period, correlated with long-term cognition repercussions generated by this heavy metal. Our data showed that Pb^2+^ exposition during lactation induced cognitive impairment at 60 PND, this parameter was evaluated by the buried food location test. In this test, the mice were able to learn the location of the food; analyzing the training curve, both control and Pb^2+^ group, decreased the time to reach the target according to the training trial (acquisition phase). However, when long-term memory was evaluated 24 h after of training, the mice of Pb^2+^ group spent more time and travelled a longer distance to reach the precise location of buried food, compared to control group, indicating impairment in memory consolidation. Several studies have shown that Pb^2+^ toxicity induce cognitive impairment and that this effect is stronger when the exposition occurs during the prenatal period or early postnatal life, than in older animals^3^. However, the particular mechanism by which this heavy metal induces motor and cognitive impairment remains elusive. This work shows the first evidence that Pb^2+^ exposure at early life, can impair brain tryptophan catabolism, which is correlated with the cognitive damage associated to this heavy metal. This study also demonstrates that Pb^2+^ induces fluctuations on KP and, considering our results, as well as previous evidence, the effect of this heavy metal on long term memory could be due to the fact that both Pb^2+^ and KYNA, interact with NMDAr. The NMDAr activation is essential during the development of neuronal networks; its inhibition during the early postnatal life reduces synchronous network activity both in neocortex and in hippocampus^15,37,38^ leading to impairment in memory functions^9^.

In this regard, it has been described that Pb^2+^ can modulate NMDAr by different routes: (1) Pb^2+^ blocks NMDAr in the synaptic region disrupting the calcium signaling mechanisms and (2) Pb^2+^ exposure enhances NR2B subunit expression of NMDA receptor in the extra synaptical area producing an increase in calcium concentration^39–41^. Regarding KYNA, it has been demonstrated that it is an endogenous competitive antagonist to glycine site of the NMDAr^42,43^, and the prenatal exposure to its immediate precursor, L-kynurenine, is associated with reduction of NR1 and NR2A expression, leading to lower NR2A/NR2B ratio^44–46^. It has also been suggested that high KYNA levels in the prenatal brain inhibit neurite branching and development of excitatory synapses; after birth, KYNA levels decline to allow excitatory synapse formation^47,48^. In our model, the excessive blocked of NMDAr in early postnatal life by KYNA and Pb^2+^, could produce alterations in different processes during neurodevelopment, resulting in long-term cognitive deficits as was observed under our experimental conditions. Alternatively, KYNA can impact brain development and cognitive processes since it is an endogenous negative allosteric modulator of α7 nicotinic acetylcholine receptors (α7nAChRs), which play critical roles in regulating neuronal plasticity^49–51^ and cellular survival in the brain^49,52–54^.

Coupled to possible structural alterations evoked by Pb^2+^ exposure and KYNA levels fluctuations during the early postnatal life, the physiopathological consequences of the variations in brain levels of KYNA found in mice at 60 PND, raise the possibility that KYNA might interact with less receptors due cell death caused by Pb^2+^, exposure, having influence in the neurotransmission and intensifying the alterations in long term memory. In light of the present results, it is likely that the high levels of KYNA may be due at least in part to the neuronal cell damage induced by Pb^2+^, which is reflected in a larger number of astrocytes in relation to neurons.

On the basis of the results showed herein, it is tempting to speculate that the cognitive impairment observed in humans exposed to Pb^2+^ is related with elevated levels of KYNA or some other KP metabolites. Further studies in our laboratory are focused to elucidate the impact of KP modulation during brain development as well as on cognitive impairment induced by Pb^2+^ toxicity.

## Conclusion

In summary, this is the first study that demonstrates the positive correlation between high levels of KYNA and cognitive impairment induced by Pb^2+^ toxicity in early postnatal life. Therefore, manipulations of the KP may represent a potential novel treatment strategy for conditions that are linked to motor and cognitive perturbations induced by this heavy metal.

## Material and Methods

### Animals

Experimental protocol (Number 109/15) was approved by the Institutional Research Committee of the National Institute of Neurology and Neurosurgery and by the Institutional Committee for the care and use of laboratory animals (CICUAL-INNN). All procedures with animals were carried out according to regulations specified by the Bioethical Committee, and the Mexican Regulation for the production, care and use of laboratory animals (NOM-159 062-ZOO-1999). During the dissections, all efforts were made to minimize animal suffering. FVB mice (28–32 g) from the animal house of the National Institute of Neurology (Mexico City) were employed throughout the study. Animals were housed in acrylic cages with standard commercial mice diet and water ad libitum. The housing room was maintained under constant conditions of temperature (25 ± 3 °C), humidity (50 ± 10%) and lighting (12 h light/dark cycles

Each dam was housed with a male mouse in individual cages until vaginal plug was detected, then the male mouse was removed from the cage. At birth, litters were assigned randomly to one of two groups: 1) Control group and 2) Pb^2+^ group. Offspring were biochemistry and behavioral tested at 23 and 60 PND by a researcher who was blind to the treatment conditions.

### Lead exposure

Dams and their offspring from control group^1^ received normal drinking water during the study, while dams and their offspring from Pb^2+^ group^2^ received Pb^2+^ in the drinking water (500 ppm of lead acetate), from the birth to 23 PND. Only males were analyzed to avoid any influence of the female hormonal system on metabolism. After weaning at 23 days of age, the male offspring from different litters were randomly housed, 2–3 per cage, for both groups and received normal drinking water to evaluate long-term effects (60 PND). For the biochemistry and behavioral evaluation, experimental subjects were the progeny from 9–11 litters per group, with no more than 3 pups from any given litter. This design was implemented in order to minimize the contribution of individual litters.

### Lead determination in brain regions of mice

Pb^2+^ levels were evaluated in different brain regions, hippocampus, striatum, cortex and cerebellum of mice (23 PND) of both groups by graphite furnace atomic absorption spectrophotometry. Tissue samples were weighted and 65% suprapure nitric acid (Merck, Mexico) (1:10, w/v) was added, then the tissue was digested in a shaking water bath at 60 °C for 30 min. Afterwards, the samples were diluted (1:10 or 1:20, v/v) with deionized water. Samples were injected into an atomic absorption spectrophotometer (Model 3110; Perkin-Elmer, Norwalk, CT, USA) equipped with graphite furnace (HGA-600) and auto-sampler (AJS60) adjusted to a wavelength of 283.3 nm. Quality control standards and calibration curves (of known amounts of Pb^2+^ standard) were used for each analysis. Results were expressed as µg of lead for mg of tissue^55^.

### Evaluation of motor activity in mice

Locomotor activity of mice from both groups at 23 and 60 PND was evaluated in an Opto-Varimex 4 system (Columbia, Ohio, USA). Briefly, the motor/kinetic activity was recorded during 5 min after the habituation period and the cage was cleaned with alcohol between each test to exclude any effect of the previous animal. The results are expressed as total distance walked (cm) and ambulatory time (sec) recorded in 5 min.

### Buried food location test (BFLT)

A researcher who was unaware of the treatment condition performed all behavioral experiments.

The buried food location test (BFLT) was performed in mice at 60 PND. BFLT is an adaptation of the model described by Lehmkuhl *et al*. for olfactory dysfunction evaluation^56^.

This version of the test comprises two sessions. The training session (acquisition) consisted of 6 trials (1–6, 2 minutes inter-trial interval) and a 0 trial in which each mouse was placed in an acrylic box (1 m^2^), covered with a 3 cm-sawdust layer and a highly palatable food (sugary pellet, “froot loops”, to which they were previously familiarized) buried 1 cm under the sawdust, in a fixed quadrant of the box. The location of the pellet in all the trials was the same and the box exhibited spatial clues (black color geometric figures of 10 cm × 10 cm, placed in the middle of each face of the box at a height of 13 cm). In case the mice were unable to find the froot-loop within 180 s in the first trial, they were gently guided to it (the froot loop should be placed on the surface). The mice were allowed to eat the froot loop for 5 s. During this session, the animals were fasted for the previous 24 hours with *ad libitum* access to water. After each trial, the mice were returned to its home cage, the testing area was cleaned with a 10% ethanol solution and the sawdust removed in order to eliminate odoriferous marks. The long-term memory was evaluated 24 hours after the acquisition session, in a 3 minutes retention test where the food was removed from the sawdust and mice were allowed to freely explore the arena for 180 s; the time spent in reaching the precise location of buried food (same location as in the acquisition) was recorded and the exploration time spent in the food location quadrant was also measured. All the sessions were video recorded allowing us to analyze the videos offline with a tracking software ImageJ, USA. The results are expressed as the distance travelled to reach the objective (cm), the time to reach the target and the time spent searching for the food in the target quadrant (sec).

### Tissues treatment

After treatments, offspring of both groups of mice were sacrificed by decapitation and brain regions were obtained (hippocampus, striatum, cortex and cerebellum). For reactive oxygen species (ROS), cellular function (MTT) and lipid peroxidation (LP) determination, brain regions were sonicated in 500 µl of Krebs buffer pH 7.4 (19 mM NaCl, 5 mM KCl, 2 mM CaCl_2_, 1.2 mM MgSO_4_, 5 mM glucose, 13 mM NaH_2_PO_4_ y 3 mM Na2HPO_4_; J.T. Baker, USA). KP metabolites were determined in the brain regions previously weighted and homogenized in deionized water (1:10, w/v). The homogenates were centrifuged at 14600 g for 10 min in a Select Bioproducts centrifuge (SB products; PO Box, Edison NJ) and the supernatant was used to inject into the HPLC.

### ROS quantification

ROS were evaluated through DCF-DA oxidation^29^. Briefly, brain regions homogenates were incubated with DCF-DA (75 μM, cat. No. 35845, Sigma-Aldrich, USA) for 30 min at 37 °C in darkness and then centrifuged at 6000 × g (SB products; PO Box, Edison NJ) for 10 minutes. After incubation, ROS formation was quantified in supernatants through fluorescence spectrophotometry in a plate reader Flx-800 (Biotek Instruments, Winooski, VT, USA) at an excitation wavelength of 448 nm and 532 nm of emission. Results were expressed as milligram of DCF per milligram protein.

### Lipid peroxidation determination

Lipid peroxidation was evaluated through production of thiobarbituric acid reactive species (TBA-RS). Briefly, 125 µl of brain regions homogenates were boiled with 250 µl of TBA (0.375 g of thiobarbituric acid (cat. No. T5500, Sigma-Aldrich, USA) + 15 g of trichloroacetic acid (TCA) + 2.54 mL of HCl in 100 ml (J.T. Baker, USA)) in a water bath during 15 min, then they were placed on ice and finally, they were centrifuged at 9800 × g (SB products; PO Box, Edison NJ) for 10 min. The optical density of supernatant was obtained in an Eon microplate reader (Biotek Instruments, Winooski, VT, USA) at a wavelength of 532 nm^57^. Results were expressed as micromoles of malondialdehyde per milligram protein.

### MTT reduction assay

Cellular function was evaluated by MTT reduction assay^57–59^. Briefly, 100 µl of homogenate of brain regions were incubated with 4 µl of MTT (5 mg/mL, cat. No. M5655, Sigma Aldrich, USA) at 37 °C during 15 min. Samples were centrifuged at 12000 × g for 3 minutes and supernatant was eliminated. 250 µl of acidified isopropyl alcohol were added to dissolve the formazan. Optical density was obtained in an Eon microplate reader (Biotek Instruments, Winooski, VT, USA) at a wavelength of 570 nm. Results were expressed as the percentage of MTT reduction in relation to control values for each brain region.

### GSH determination

Tissue GSH levels were determined in four brain regions using an adapted fluorescence method with o-phthalaldehyde; the assay was performed in a microplate where the GSH reacts with o-phthaldialdehyde (OPA) forming a highly fluorescent isoindole derivative^60^. Brain regions were homogenized (1:10 w/v) in buffer A (154 mM KCl, 5 mM diethylenetriaminepenta-acetic acid (DTPA) and 0.1 M potassium phosphate buffer pH 6.8 proteases inhibitor and PMSF). Then, it was added cold B buffer (40 mM HCl, 10 mM DTPA, 20 mM ascorbic acid and trichloroacetic acid 10%). The tissues were centrifuged at 14000 × g during 20 min. Then, 5 µl of supernatant was added with OPA to obtain the isoindole. The fluorescence was measured at 370 nm of excitation and 420 nm of emission in a microplate lector (Flx-800, BioTec). The results were expressed as nanomoles of GSH/g of tissue.

### Kynurenic acid (KYNA) determination

KYNA levels were determined in brain regions of mice of 2 different ages by an HPLC method with fluorescence detection (model S200). 20 µl of sample or standard solution were injected into Eclipse XDB-C18 reverse phase column (5-μm, 4.6 × 150 mm, Agilent, Santa Clara, CA, USA) and isocratically eluted with a polar mobile phase (250 mM zinc acetate (cat. No. 2740, Meyer, Mex), 50 mM sodium acetate (cat. No. 79714, Sigma-Aldrich, USA), pH 6.2 adjusted with glacial acetic acid (J. T. Baker, USA) and 3% of acetonitrile (HPLC grade, Sigma- Aldrich, USA). Samples were eluted at a flow of 1.0 ml/min. KYNA was detected by fluorescence at emission wavelength of 398 nm and 344 nm of excitation wavelength^35^. Retention time was ~7 min. The results were presented as fmoles of KYNA/mg of protein. The results were presented as fmoles KYNA/mg of protein.

### Kynurenine aminotransferase (KAT-II) activity

KAT-II activity was measured in brain regions of mice of 23 and 60 days old, which were homogenized (1:10, v/v) in homogenization buffer (containing 0.5 M tris-base buffer pH 8 (J. T. Baker, USA), 50 µM of pyridoxal-5-phosphate (P5P) and 10 mM 2-mercaptoethanol (cat. No. P9855, M6250 Sigma-Aldrich)). 100 µl of homogenized tissues were incubated with 100 µl of cocktail (containing 100 µM of L-kyn (cat. No. K8625, Sigma-Aldrich), 80 µM of P5P, 1 mM of sodium pyruvate (cat. No. P2256, Sigma-Aldrich) containing 150 mM tris-base buffer pH 7.4) and incubated at 37 °C for 1 h. The reaction was stopped with 20 µl of trichloroacetic acid 50% and 1 mL of HCl 0.1 M (J.T. Baker, USA). Samples were centrifuged 10 min at 14000 g. KYNA was detected by fluorescence at emission wavelength of 398 nm and 344 nm of excitation wavelength (Perkin Elmer fluorescence detector series 200a)^61^. The results were presented as pmoles of KYNA/h/mg of protein.

### 3-hydroxykynurenine (3-HK) determination

3-HK levels were determined in brain regions (cortex, hippocampus, striatum and cerebellum) by HPLC using an electrochemical method^62^. Briefly, 40 µl of supernatant were eluted at a constant flow rate of 0.5 ml/min with a mobile phase (950 ml deionized water, 5.9 ml phosphoric acid (J.T. Baker, USA), 20 ml acetonitrile (HPLC grade), 9 ml triethylamine, 100 mg sodium EDTA, and 1.2 g heptane sulphonic acid (cat. No. T0886, E1644 and H2766, Sigma-Aldrich, USA)) through an Adsorbosphere Catecholamine C18 reverse phase column (3-μm, 4.6 mm × 100 mm, Fisher Scientific, Hampton, Nuevo Hampshire, USA). Oxidation voltage was 0.500 V, at a range 1.0 nA and a filter of 0.10 Hz (LC-4C detector, BAS). The retention time was ~11 min. The results were presented as pmoles of 3-HK/mg of protein.

### Kynurenine monooxygenase (KMO) activity

KMO activity was measured in brain regions homogenates. 100 µl of homogenized tissues were diluted in 400 µl of KMO buffer activity (containing 100 mM TRIS, 10 mM KCl and 1 mM EDTA, Sigma-Aldrich, USA). Then, 80 µl of homogenate were incubated with 100 µl of assay cocktail (1 mM of NADPH, 3 mM of G6P, 1 U/ml of G6PDH and 100 µM of L-kynurenine sulfate (cat. No. N1630, G6378 and K2750 Sigma-Aldrich respectively)) and incubated at 37 °C for 1 h. The reaction was stopped with 25 µl of perchloric acid 6%. Samples were centrifuged 10 min at 14000 g. 40 µl of supernatants were injected to HPLC and 3HK levels were detected with the above mentioned method^62^. The results were presented as pmoles of 3-HK/h/mg of protein.

### Protein determination by Lowry

Protein quantification was evaluated through Lowry methodology, 10 µl of homogenized tissue was diluted with 190 µl of water. It was added 1 ml of C solution (A solution: Na_2_CO_3_ 2%, NaOH 0.4% and 0.2% of sodium tartrate + B solution: Cu(SO_4_)_3_ 0.5% Sigma-Aldrich, USA). Samples were incubated 10 min at room temperature and then Folin reactive (50%, cat. No. F9252, Sigma Aldrich, USA) was added and mixed. They were incubated for 30 min at room temperature. Absorbance was determined at 550 nm in a plate reader (Eon Biotek Instruments, Winooski, VT, USA)^63^.

### Percentage of KAT and KMO positive cells and expression in brain by flow cytometry

Expression of the kynurenine pathway enzymes was determined in mice brains from lead treated and control groups at 60 PND. Mice brains were washed with cold PBS 1Χ and cut into small pieces, then 1 ml of accutase (cat. No. L0950, BioWest) was added. Accutase was inactivated with DMEM/F-12 (cat. No. R36/37/38, BioWest) supplemented with 10% FBS (cat. No. S1650, BioWest). Disaggregated brains were filtered in 0.7 mm pore filters, cells were centrifuged at 200 × g for 5 minutes, the supernatant was discarded, and cells were washed twice. After this, cells were resuspended in 30% Percoll (cat. No. 17-0891-01, GELife Sciences) and then centrifuged at 200 × g for 20 minutes. Following centrifugation, the myelin layer was totally discarded, cells were washed with PBS 1Χ and centrifuged. After washing, cells were resuspended with FACS permeabilizing solution (cat. No. 340973, BD FACS), and incubated during 30 minutes at room temperature. After incubation, cells were washed with PBS 1Χ and centrifuged. Then, cells were resuspended with mouse anti-KAT (0.1 µg/ml cat. No. B0408 SC-67376, Santa Cruz Biotechnology) or anti-KMO (0.1 µg/ml cat. No. OAAB05255, AVIVAS) and incubated during 30 minutes at room temperature. Following incubation with the primary antibodies, cells were washed with PBS 1Χ, centrifuged and then, resuspended with Alexa Fluor 594 anti-rabbit IgG (0.1 µg/ml cat. No. A11012, Molecular Probes), cells were incubated during 30 minutes at room temperature at darkness. After incubation, cells were washed with PBS 1Χ and fixed with 1% paraformaldehyde. Percentage of positive cells and mean fluorescence intensity (MFI) was determined by flow cytometry using a BD FACSCalibur FL4 instrument (BD Biosciences), analysis was done using CellQuest and Flowjo v.10 software.

### Histology assay

The mice (60 PND) were deeply asleep with pentobarbital overdoses and perfused transcardiacally with chilled 10% w/v buffered formalin solution. The whole brains were removed immediately after perfusion and fixed for histological examination into 10% formaldehyde solution for 24 h, dehydrated and embedded in paraffin. Coronal sections of 5 µm thickness were cut with a microtome and stained with hematoxylin–eosin. Hippocampus sections were evaluated using the mouse brain atlas and stained to determine cell damage through morphology, including neurons with a normal appearance, or identifying those showing signs of damage (hyperchromatic cytoplasm, cellular shrinking, and nuclear fragmentation). The histopathology analysis was performed by independent pathologist blind to the experimental conditions. Three slides with 10 fields per mice were analyzed under light microscopy for morphometric analysis; the digitization and storage of images were performed with a light microscope Axio Lab A1 Zeiss equipped with Axicocam ICC5 camera at a 1000X magnification An unbiased counting frame was placed directly onto the screen of the PC over the reference section and the section under analysis, and counting was performed using basic principles of the optical dissector counting method reported by West (1993). In the optical dissector, the cells were counted only if a feature of interest was clearly recognized within the dissector height and located inside the counting frame or touching the inclusion line. Neurons touching the exclusion line were not counted. The percentages of damaged cells were obtained by dividing the number of damaged cells between number of total cells in the ten fields counted in each slide, and the total number of cells was expressed as the percentage of cells per field^64^.

### Data analysis

Data were corrected by mg of protein. Results were expressed as mean values ± S.E.M. Both T-test and Mann-Whitney test were used to compare distributions of Control and Pb^2+^ groups using the Prism software (GraphPad, San Diego, CA, USA). Values of p < 0.01 were considered statistically significant. Additionally, the correlation between each pair of the following variables was assessed using the Spearman’s rho coefficient: hippocampus KYNA levels, time to reach the target and distance traveled to reach the objective.

## Data Availability

Data used to support the findings of this study are available with the corresponding author upon request.
